# The Association between the Lipids Levels in Blood and Risk of Age-Related Macular Degeneration

**DOI:** 10.3390/nu8100663

**Published:** 2016-10-22

**Authors:** Yafeng Wang, Mingxu Wang, Xiaoqing Zhang, Qianyu Zhang, Jing Nie, Ming Zhang, Xiaohong Liu, Le Ma

**Affiliations:** 1 The First Affiliated Hospital, Xi’an Jiaotong University College of Medicine, Xi’an 710061, China; wyf.90.25.wyf@stu.xjtu.edu.cn; 2 Key Laboratory of Shaanxi Province for Craniofacial Precision Medicine Research, College of Stomatology, Xi’an Jiaotong University, Xi’an 710004, China; 3 School of Public Health, Xi’an Jiaotong University Health Science Center, Xi’an 710049, China; wangmx601@xjtu.edu.cn (M.W.); MICHELLE921019@stu.xjtu.edu.cn (Q.Z.); 4 Department of Public Health, Xi’an Medical University, Xi’an 710021, China; dyzhou@nwpu.edu.cn; 5 School of Humanities, Xi’an Jiaotong University, Xi’an 710049, China; boraisrighthere@sina.com; 6 Xi’an Honghui Hospital, Xi’an 710054, China; 7 Key Laboratory of Environment and Genes Related to Diseases (Xi’an Jiaotong University), Ministry of Education of China, Xi’an 710049, China

**Keywords:** age-related macular degeneration, blood lipids levels, HDL, meta-analysis

## Abstract

Lipid metabolism may be involved in the pathogenic mechanism of age-related macular degeneration (AMD). However, conflicting results have been reported in the associations of AMD with blood lipids. We performed a meta-analysis including a total of 19 studies to evaluate associations between blood lipids and this disease. The result reported that the high level of high-density lipoprotein cholesterol (HDL-C) obtained with an increment of 1 mmol/L could result in a significantly increase in the AMD risk of approximately 18% (relative risk (RR), 1.18; 95% confidence interval (CI), 1.01 to 1.35; *I*^2^ = 53.8%; *p* = 0.007). High levels of total cholesterol (TC), low-density lipoprotein cholesterol (LDL-C), and triglycerides (TG) were significantly associated with a decreased risk of AMD (RRs ranging from 0.92 to 0.95; all *p* < 0.05). The stratified analysis based on AMD subtypes showed that these blood lipids were only significantly associated with the risk of early AMD (all *p* < 0.05). The association between the blood lipids and AMD risk did not differ substantially based on the other characteristics of the participants. A high HDL-C level was associated with an increased AMD risk, whereas participants with high TC, LDL-C, and TG concentrations may show a decreased risk for this disease. Further well-designed large studies are warranted to confirm the conclusions.

## 1. Introduction

Age-related macular degeneration (AMD) is a leading cause of irreversible vision loss among people aged 65 and older in western countries [1]. In fact, 8.7% of the worldwide population has AMD, and the projected number of people with this disease is projected to increase to approximately 196 million in 2020, and to 288 million in 2040 [2]. A substantial increase in aging populations makes this disease have a significant public health impact, and the burden of AMD is increasing [3]. The pathological hallmark of early stage AMD is large drusen and pigmentary abnormalities due to deposits of lipids and proteins in the retinal pigment epithelium. Progression to late stage is characterized by the occurrence of severe visual impairment either as a result of choroidal neovascularization (CNV) with hemorrhage or through atrophy of the retinal pigment epithelium (RPE) and photoreceptor cells [4].

Although the pathogenic mechanism remains elusive, the deposition of lipoproteins has been implicated in the pathogenesis of AMD and atherosclerosis [5]. A high level of high-density lipoprotein cholesterol (HDL-C) could induce reverse cholesterol transport and improve endothelial function, which decreases the risk of atherosclerosis [6], whereas a high level of low-density lipoprotein cholesterol (LDL-C) exerts effects on atherosclerosis [7]. Previous epidemiological studies that evaluated the blood lipids in AMD have not yielded similar relationships, and some studies have shown inverse relationships between these lipid levels and AMD risk [8,9,10]. Furthermore, some of the HDL-increasing alleles in the HDL-C pathway, such as the lipoprotein lipase gene, the cholesterol ester transferase gene, and the ABC-binding cassette A1 gene, have been associated with increased AMD risk [11]. In addition, dyslipidemia has been closely associated with the formation of drusen, which are likely to develop into early AMD [12], but it is unclear whether the associations of the blood lipids are different at different stages of this disease.

Therefore, we conducted a meta-analysis pooling the results of all available association studies between these lipids concentrations and the risk of AMD. Furthermore, a stratified analysis was also performed to examine the associations of these lipids with differentiation in AMD subtype.

## 2. Materials and Methods

This meta-analysis was performed in accordance with Preferred Reporting Items for Systematic Reviews and Meta-Analyses (PRISMA) guidelines.

### 2.1. Data Sources and Search Strategy

We conducted a comprehensive search of the PubMed, EMBASE, and ISI Web of Science databases from their inception to January 2016 for relevant studies that estimated the association between serum lipids and risk of AMD using the following search terms: (Cholesterol or lipids or high-density lipoprotein or HDL or low-density lipoprotein or LDL or triglycerides or triglycerides (TG)) and (AMD or age-related maculopathy or neovascular AMD or exudative AMD or choroidal neovascularization or geographic atrophy or macular degeneration). The search was not restricted to any language. In addition, the reference lists of the retrieved articles were subsequently evaluated to identify any studies not identified from the preliminary literature searches. When necessary, the corresponding authors were contacted for additional information.

### 2.2. Study Selection

All of the studies evaluating the associations of total cholesterol (TC), high-density lipoprotein cholesterol (HDL-C), low-density lipoprotein cholesterol (LDL-C), and TG levels with AMD risk were potentially included. The following criteria were included for the selection: cohort, case-control, or cross-sectional studies published as an original article; the major objective of the study included in the meta-analysis was to evaluate the relationship between these blood lipids and any types of AMD; the information on the type of AMD (early AMD or late AMD (wet or dry)) was assessed in the study; and the relative risks (RRs) [or odds ratios (ORs) or hazard ratios (HRs)] with 95% confidence intervals (CIs) or sufficient data to calculate these parameters were reported. Main exclusion criteria for a study were as follows: the study was a review or editorial comment; the study could not provide sufficient information; the study was not regarding AMD research. When multiple publications reported the same or overlapping data, the study with the largest sample size was included in the analysis. Two investigators (Yafeng Wang and Mingxu Wang) independently reviewed the retrieved records. Any inconsistencies were resolved through consensus with a third author (Le Ma) for adjudication.

### 2.3. Data Extraction and Study Quality Assessment

The following characteristics of each of the identified studies were collected: first author, publication year, study name, country, study type, sample size, mean age, sex, sample source, exposure, classification criteria outcomes, diagnosis method, type of AMD, and covariates in a fully adjusted model. Data on different AMD subtypes were extracted; moreover, the RRs of not only total AMD but also the AMD subtypes (early or late AMD) were all abstracted. Furthermore, geographic atrophy (GA) or CNV were also extracted for late AMD. When the studies only provided subcategories of the AMD disease status, the gratings were collapsed into a single AMD group.

The quality of the included studies was independently appraised with the Meta-analysis Of Observational Studies in Epidemiology (MOOSE) guidelines [13]. When less than half of the criteria were clearly described and accounted for, the studies were considered low quality, and, the studies that met at least half of the criteria were considered high quality. The information was carefully extracted, and two investigators (Yafeng Wang and Mingxu Wang) independently assessed the study quality. Any disagreements were settled through consensus.

### 2.4. Statistical Analysis

A RR with 95% CI is a commonly used measure of effect of interest in the medical and public health literature. When HRs and incidence risk ratios were reported, these parameters were directly considered RRs. Because AMD is not common, we therefore reported ORs as equivalent to RRs. The generalized linear models were used in many studies and therefore we assumed a linear relation between exposure and outcome. RRs were expressed for a standardized increase in the lipid concentrations of 1mmol/L which is the most frequently measure. All lipid concentrations were converted, if necessary, to 1mmol/L. For studies that reported results separately by gender, AMD stage, or race, the risk estimates were pooled using a fixed-effects model before the study was included in the overall analysis. According to the heterogeneity, a random-effects model or fixed-effects model was used in our meta-analysis. The heterogeneity among individual studies was evaluated by calculating the Cochran’s Q statistic and the *I*^2^ test (*I*^2^ > 50% was considered high). In cases of potential heterogeneity, a meta-regression was performed to explore the potential sources of heterogeneity between studies. Subgroup analysis was conducted based on study type (cohort v. cross-sectional), ethnicity (America vs. Europe vs. Australia vs. Asian), classification criteria of AMD [International Classification and Grading system (ICGS) vs. Wisconsin Age-related Maculopathy Grading System (WARMGS) vs. Age-Related Eye Disease Study (AREDS)], type of AMD (early or late AMD (GA/CNV)), mean age of participants (≥65 vs. <65 years), and study quality (high vs. low). In addition, sensitivity analyses were performed, after sequentially removing one study at a time to evaluate the stability of the results. Begg’s test, Egger’s test, and funnel plots were evaluated to assess for publication bias risk [14,15]. We used STATA version 11.0 (Stata Corp LP, College Station, TX, USA) for all statistical analyses. Two-sided *p* values less than 0.05 were considered statistically significant, and in Egger’s linear regression and Begg’s rank correlation, a level of 0.10 was used.

## 3. Results

### 3.1. Literature Search

**Figure 1:**
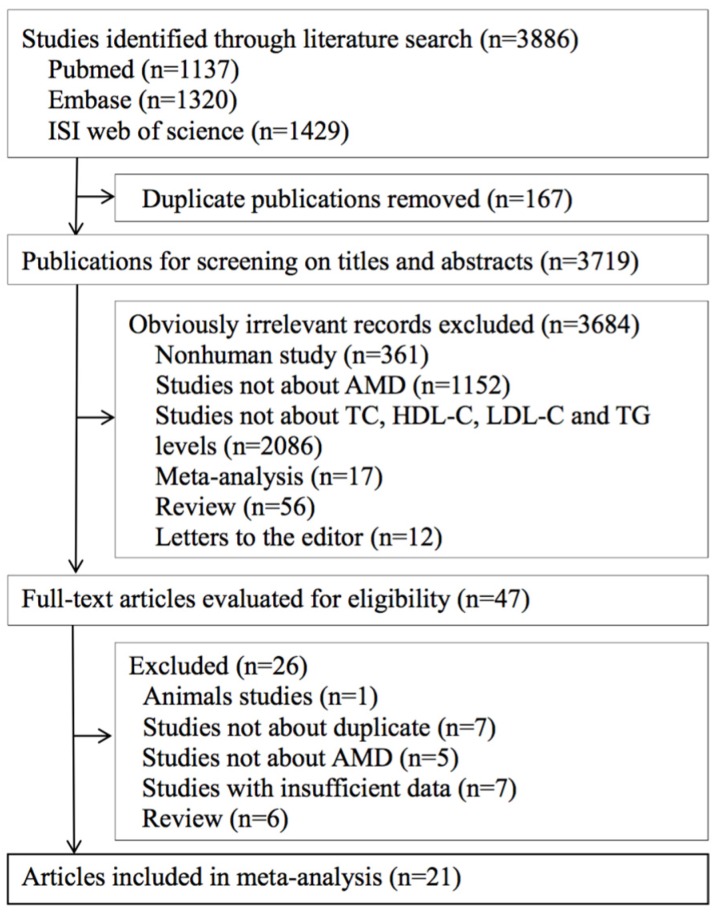
Flowchart for the selection of eligible studies. AMD, age-related macular degeneration.

A total of 3886 articles were retrieved from the search, and 47 potentially relevant studies were eligible for further review. After reviewing the full-text articles, 21 articles (19 studies) were included in this meta-analysis (Figure 1) [8,9,10,16,17,18,19,20,21,22,23,24,25,26,27,28,29,30,31,32,33].

### 3.2. Study Characteristics

The main characteristics of the studies are described in Table 1. Among the 19 studies, 6 studies were cohort studies and 14 studies were cross-sectional studies. The number of subjects included 82,966 participants, ranging from 163 to 14,752. Among these studies, seven studies were conducted in Europe, six studies were conducted in Asia, five studies were conducted in America, and one study was conducted in Australia. The study population in 18 studies included both men and women, and 1 study consisted entirely of men. The average age of the subjects ranged from 49.0 to 78.5 years. The diagnosis of AMD was based on fundus photography in all studies, with the exception of one study that did not report the AMD diagnosis. Fourteen studies used the WARMGS criteria to establish AMD, whereas the ICGS criteria and AREDS criteria were applied in four studies and one study, respectively. Most of the studies were adjusted for age (*n* = 18), gender (*n* = 14) and smoking (*n* = 10), whereas a fewer number of adjusted for body mass index (*n* = 6), hypolipidemic drug use (*n* = 3), and alcohol consumption (*n* = 2).

### 3.3. HDL-C Level and AMD Risk

**Figure 2:**
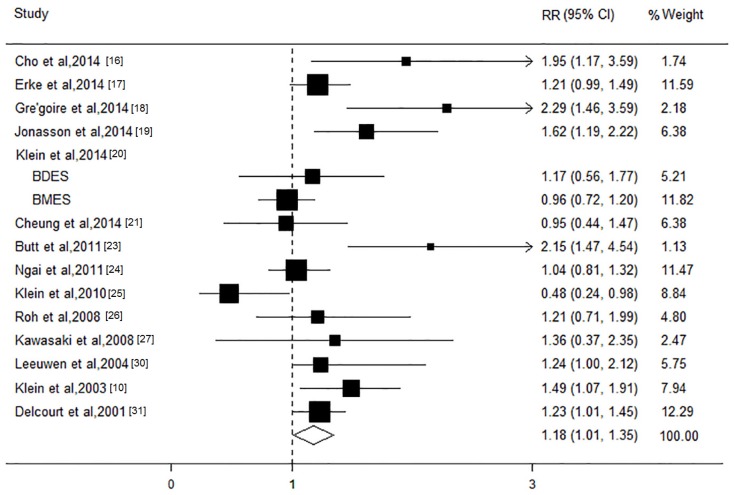
Forest plot on the associations between the high-density lipoprotein level and age-related macular degeneration. The boxes and lines indicate the relative risks (RRs) and their 95% confidence intervals (CIs) on a log scale for each study. The pooled relative risk is represented by a diamond. The size of the black squares indicates the relative weight of each estimate. BDES, the Beaver Dam Eye Study; BMES, the Blue Mountains Eye Study.

The relationship between the HDL-C level and risk of AMD was evaluated in 15 studies, comprising 53,981 participants. Among these, most studies showed inverse associations between a higher HDL-C level and AMD risk, and significant associations were observed in seven studies. The results of the present meta-analysis revealed that an elevation in the HDL level of 1 mmol/L increment could result in a significant increase in the AMD risk of approximately 18% (RR, 1.18; 95% CI, 1.01 to 1.35; *I*^2^ = 53.8%; *p* = 0.007; Figure 2). The results of stratified analysis based on cohort studies found that the per 1 mmol/L increment of HDL level had a tendency to increase the risk of AMD, but did not reach statistic significant (RR, 1.08; 95% CI, 0.93 to 1.24; Table 2). The grouping of the studies by AMD stages showed that an increment of 1 mmol/L in the HDL-C level was associated with a 10% increase in the risk for early stage (RR, 1.10; 95% CI, 1.01 to 1.19), but not late stage AMD (RR, 1.14; 95% CI, 0.81 to 1.46). In the subgroup analysis based on late AMD subtypes, the pooled RRs for an increase in the HDL level of 1 mmol/L were 1.06 (95% CI, 0.51 to 1.62) and 0.99 (95% CI, 0.23 to 1.75) for CNV and GA, respectively. The stratified analysis across a number of participant characteristics showed that these factors did not significantly alter the shape of the association of the HDL-C level with AMD risk. In addition, sensitivity analyses showed that the study conducted by Klein et al. was the main source of heterogeneity. After excluding this study from the analysis, the potential heterogeneity substantially decreased to 26.6% for any AMD; however, the exclusion of this study from the pooled estimate had little impact on the overall effect size (RR, 1.18; 95% CI, 1.08 to 1.29; *I*^2^ = 26.6%; *p* = 0.17). Moreover, the results of sensitivity analysis suggested that no single study could influence the overall pooled estimates. Begg’s funnel plot and Egger’s regression test did not reveal any evidence of the presence of publication bias in the eligible studies (both *p* > 0.1).

### 3.4. LDL-C Level and AMD Risk

**Figure 3:**
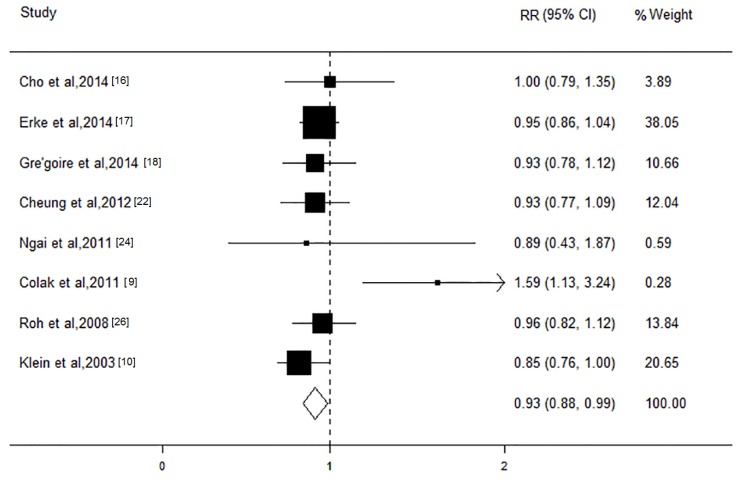
Forest plot on the associations between the low-density lipoprotein level and age-related macular degeneration. The boxes and lines indicate the relative risks (RRs) and their 95% confidence intervals (CIs) on a log scale for each study. The pooled relative risk is represented by a diamond. The size of the black squares indicates the relative weight of each estimate. BDES, the Beaver Dam Eye Study; BMES, the Blue Mountains Eye Study; SMES, Singapore Malay Eye Study; SIES, Singapore Indian Eye Study; SCES, Singapore Chinese Eye Study.

We evaluated the association of the LDL-C level with AMD in 10 studies with a total of 27,668 participants. With the exception of the study conducted by Colak et al. [9], all of these studies showed a protective tendency. The pooling of these studies in the meta-analysis yielded an RR of the LDL level of 0.93 (95% CI, 0.88 to 0.99; *I*^2^ = 0; *p* = 0.83; Figure 3) for an increment of 1 mmol/L, without heterogeneity. The stratified analysis based on AMD subtypes showed that increase in the LDL level of 1mmol/L had an apparently protective effect on early stage (RR, 0.95; 95% CI, 0.88 to 0.99; *I*^2^ = 0; *p* = 0.99), but not on late stage (RR, 1.00; 95% CI, 0.86 to 1.13; *I*^2^ = 18.9; *p* = 0.30). The subgroup analysis revealed that most of the other characteristics shared consistency in the direction of the effect. In addition, the sensitivity analysis showed that the significant relationships of the pooled RRs remained stable. We did not observe any evidence of publication bias (Begg’s test *p* = 0.66; Egger’s test *p* = 0.45).

### 3.5. TC Level and AMD Risk

**Figure 4:**
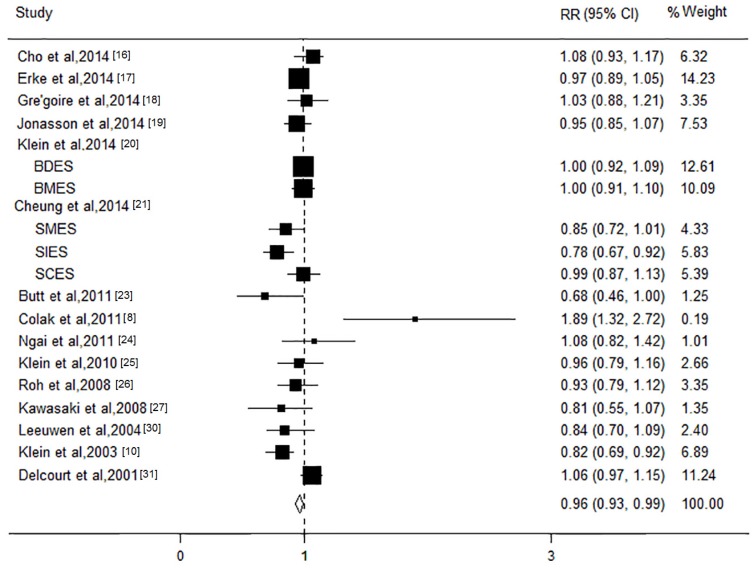
Forest plot on the associations between the total cholesterol level and age-related macular degeneration. The boxes and lines indicate the relative risks (RRs) and their 95% confidence intervals (CIs) on a log scale for each study. The pooled relative risk is represented by a diamond. The size of the black squares indicates the relative weight of each estimate.

The association between the TC level and AMD was investigated in 18 studies with a total of 54,862 participants. Most of these studies showed a protective tendency, and two studies showed a significantly increased risk of this disease. The pooled results revealed that the participants with an increase in a high TC level of 1 mmol/L were at decreased risk for AMD (RR, 0.96; 95% CI, 0.93 to 0.99; *I*^2^ = 58.9%; *p* = 0.001; Figure 4). The stratified analysis based on cohort found that increased TC level per 1mmol/L had a protective tendency for the risk of this disease (RR, 0.98; 95% CI, 0.93 to 1.03). The association was only significant (RR, 0.95; 95% CI, 0.92 to 1.00; *I*^2^ = 46.2%; *p* = 0.05) for early stage, but not for late stage AMD (RR, 0.97; 95% CI, 0.88 to 1.06; *I*^2^ = 0; *p* = 0.56). No associations were observed for subtypes of late stage AMD (CNV: RR, 1.05; 95% CI, 0.84 to 1.26; GA: RR, 0.81; 95% CI, 0.62 to 1.00). The significant heterogeneity decreased after the stratified analysis by AMD subtypes. The results of subgroup analysis showed that the associations between TC level and risk of AMD did not significantly differ according to these characteristics. Furthermore, we performed sensitivity analyses and confirmed the robustness of the results. No significant publication bias was observed (all *p* > 0.05).

### 3.6. TG Level and AMD Risk

**Figure 5:**
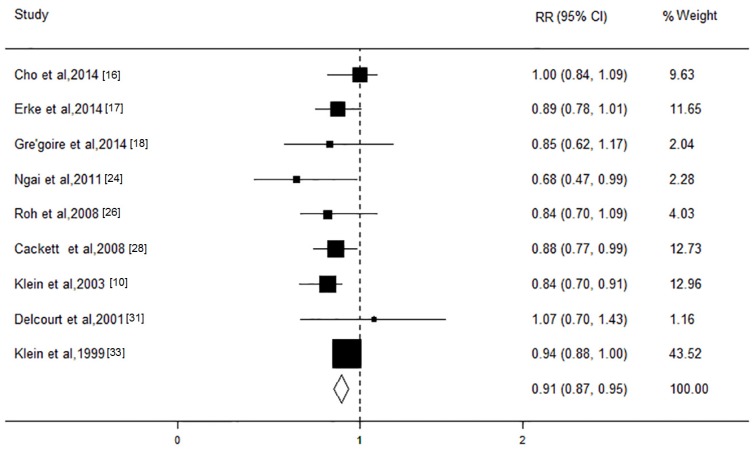
Forest plot on the associations between the triglycerides level and age-related macular degeneration. The boxes and lines indicate the relative risks (RRs) and their 95% confidence intervals (CIs) on a log scale for each study. The pooled relative risk is represented by a diamond. The size of the black squares indicates the relative weight of each estimate.

Subsequently, the association of TGs with AMD was evaluated in nine studies, with a total of 38,467 participants. The results revealed that an increase in the TG level of 1 mmol/L would significantly reduce the risk of AMD (RR, 0.91; 95% CI, 0.87 to 0.94; *I*^2^ = 2.6%; *p* = 0.42; Figure 5). The grouping of the studies according to AMD stages revealed that an increase in the TG level of 1 mmol/L would result in a significant decrease in the risk for early stage AMD of approximately 9% (RR, 0.91; 95% CI, 0.87 to 0.95; *I*^2^ = 0; *p* = 0.62) but not for late stage AMD (RR, 0.96; 95% CI, 0.82 to 1.11; *I*^2^ = 11.3%; *p* = 0.34). The results of the subgroup analysis based on these characteristics presented consistent effect directions. The sensitivity analysis showed that the pooled RRs remained stable. There was no evidence of publication bias (all *p* > 0.05).

## 4. Discussion

The results of this meta-analysis showed that elevated HDL-C could be positively associated with an increased risk of AMD, whereas high TC, LDL-C, and TG levels might have a protective role in the reduction of AMD risk. In contrast to atherosclerosis, these blood lipids showed inverse relationships with AMD, indicating that these two diseases may share different pathogeneses. In addition, the results of a stratified analysis showed that these associations were only significant for the early stage of this disease.

The deposition of blood lipids plays an important role in the pathogenesis of AMD and atherosclerosis [5,34]. Epidemiological studies have shown that a high HDL-C level is associated with a decreased risk of atherosclerosis [35]. HDL-C has been shown to induce reverse cholesterol transport associated with plaque regression and improved endothelial function. In addition, HDL-C may have anti-inflammatory and anti-thrombotic properties, which could directly affect endothelial function [36]. Therefore, a high HDL-C level could strengthen endothelial function and decrease the risk of atherosclerosis. Recent studies investigating the relationship between the HDL-C level and AMD risk have shown a positive association of a high HDL-C level with an increased AMD risk [19,30,31]. Consistent with these previous findings, the results of the present study showed that a high level of HDL-C was significantly associated with an increased risk of AMD. The adverse effect of a high HDL-C level on AMD may partially reflect the dysfunction of HDL. The macula in the central region of the retina is constantly exposed to light, and high levels of oxygen provide a favorable environment for the generation of reactive oxygen intermediates [37,38]. Under these circumstances, elevated HDL is converted into dysfunctional pro-oxidant and pro-inflammatory particles that impair cholesterol efflux and promote LDL oxidation in the retinal pigment epithelium (RPE). The protective functions of HDL would be overwhelmed through inflammation and other factors, such as myeloperoxidase-mediated oxidation, and the antioxidant and anti-inflammatory activities of HDL could become ineffective [39,40,41]. Consequently, oxidation products, such as peroxidation lipids, gradually accumulate in the retina and Bruch’s membrane where these molecules initiate inflammation and lead to amorphous deposits called drusen, which contribute to the development of AMD. Because drusen are a major pathological hallmark of early stage AMD, these associations are only significant during the early stage.

Lipid metabolism and regulation, particularly high-density lipoprotein (HDL), is complicated and involves multiple genes which may also be associations with AMD. Recently, two genome-wide association studies identified that several genetic loci including rs493258 and rs10468017 (LIPC), rs3764261 (CETP), rs1883025 (ABCA1), and rs12678919 (LPL) were heavily implicated in HDL metabolism and might have the associations with AMD [11,42]. As the key HDL metabolic pathway genes, the ABCA1 rs1883025 variant could mediate reverse cholesterol transport activity and facilitate the transfer of phospholipids and cholesterol to the liver for hepatic uptake, and the LIPC rs493258 and rs10468017 variants could encode hepatic triglyceride lipase expressed in the liver and catalyze HDL partly converted to LDL, which would both reduce the levels of HDL, retard the pro-oxidant and pro-inflammatory effect of dysfunctional excessive HDL, and lower the risk of having AMD, whereas the CETP rs3764261 variant has been demonstrated to induce CETP expression to shuttle triglyceride particles from low-density lipoproteins LDL to HDL increase the relative levels of triglyceride-enriched HDL and ultimately lead to the increased risk of AMD [43,44]. Although the LPL rs12678919 variant could increase the HDL levels through the synthesis and degradation of HDL, only a critical effect on AMD risk was found.

As another risk factor for atherosclerosis, LDL-C, was found to play a protective role in the development of AMD in the present meta-analysis. The possible mechanism of action for the different effects of LDL-C on these two diseases may contribute to discrepancies in the origin of the lipoproteins. LDL-Cs in drusen and Bruch’s membrane are derived from lipids locally produced in the RPE, but not directly deposited from the circulation [38]. Alternatively, the incubation of RPE cells in the presence of high levels of LDL-C down regulate the LDL receptors, which could retard LDL in the extracellular matrix [45,46]. The low LDL-C level of the extracellular matrix could reduce the amount of lipoproteins normally deposited in Bruch’s membrane and drusen, which ultimately may contribute to a reduction in the risk for AMD. Thus, carefully designed studies are required to substantiate this association and determine the concrete mechanism.

Some potential limitations of the present study should also merit consideration in interpreting the findings. First, although the present results showed that lipids levels may significantly affect the risk of AMD, the results based on cohort studies found that lipids levels only had a tendency for the risk of this disease which was attributed to the fact that the number of cohort studies was relatively limited. Therefore, such associations still need to be investigated to confirm in further well-designed large prospective studies; Second, there is a lack of access to original source data and we are unable to make full use of time-to-event data, therefore the potential bias and confounding effects cannot be completely ruled out; however, the combined sample size was relatively large, and the present results remained robust, adding to the strength of this analysis; Third, even though most of the studies incorporated in the meta-analysis had adjusted for several confounding factors, most studies could not consider genetic susceptibility and lipid lowering medication, and the possibility these confounders might affect the AMD risk interactively with lipid metabolism could not be excluded in the present study. Therefore, further large research studies that allow for the adjustment by more confounding factors, including the genes and environmental factors, especially hypolipidemic drugs and lipid metabolism genes, should be conducted; Fourth, because of the different countries and periods in which the studies were performed, the validated classification and grading systems for AMD were inconsistently applied between studies. Although the stratified analysis showed that different separate criteria of AMD did not alter the direction of the results, such variation may also be likely to lead to an underestimation or overestimation of the association; Finally, the potential publication bias was also a concern. Although we did not observe any apparent publication bias in our statistical tests, it was still difficult to completely rule out this problem.

## 5. Conclusions

In summary, the results of the present study demonstrated that the participants with high HDL levels may be at increased risk for AMD, whereas higher levels of TC, LDL, or TG had the tendency for a decreased risk of AMD, especially for the early stage of this disease. These results suggest that lipid accumulation may have different pathogenesis in the development of atherosclerosis and AMD. However, the number of cohort studies which have the best epidemiologic evidence was the relatively limited, indicating that further well-designed large prospective studies are needed before definitive conclusions regarding the potential associations between these lipid levels and the risk of AMD can be drawn.

## Figures and Tables

**Table 1:** Characteristics of studies included in this meta-analysis of blood lipids and age-related macular degeneration.

Source	Ethnicity	Study Design	Sample	Sex (% male)	Mean Age	Blood Lipid Indicators Associated with AMD	Classification Criteria	Diagnosis Method	AMD Subtype	Study Quality *	Adjustment
Cho et al., 2014 [16]	Asian	Cross-sectional	7899	43.3	59.0	TC, HDL, LDL, TG	ICGS	Fundus photography	Early and late AMD	High	Age
Park et al., 2014 [8]	Asian	Cross-sectional	14352	48.3	58.2	TC, HDL, LDL, TG	ICGS	Fundus photography	Early and late AMD	High	Age, gender, and smoking status
Erke et al., 2014 [17]	European	Cross-sectional	2631	42.5	72.8	TC, HDL, LDL, TG	ICGS	Fundus photography	Early AMD	High	Age, gender, smoking, and systolic blood pressure
Gre’goire et al., 2014 [18]	European	Cross-sectional	963	38.1	80.2	TC, HDL, LDL, TG	ICGS	Fundus photography	Early and late AMD	High	Age, gender, educational level, smoking, BMI, hypertension, hypolipidemic drug, cardiovascular disease, diabetes, ApoE2, ApoE4, CFH Y402H, ARMS2 A69S, LIPC(rs10468017), LIPC(rs493258) LPL, ABCA1 and CETP polymorphisms
Jonasson et al., 2014 [19]	European	Cohort	2868	42.4	74.7	TC, HDL	WARMGS	Fundus photography	Early and late AMD	High	Age and gender
Klein et al., 2014 [20]	American/European/Australian	Cohort	6953	43.5	63.4	TC, HDL	WARMGS	Fundus photography	Early and late AMD	High	Age, sex, body mass index, history of smoking status, diabetes status, hypertension status, and statin use
Cheung et al., 2014 [21]	Asian	Cross-sectional	9799	NR	54.0	TC, HDL, LDL	WARMGS	Fundus photography	Early and late AMD	High	Age, gender, body mass index, hypertension, diabetes, current smoker, alcohol consumption, chronic kidney disease, and race
Cheung et al., 2012 [22]	Asian	Cross-sectional	3172	49.2	53.7	TC, HDL, LDL	WARMGS	Fundus photography	Total AMD	High	Age and race
Butt et al., 2011 [23]	American	Cross-sectional	1019	39.9	62.4	TC, HDL	WARMGS	Fundus photography	Total AMD	High	Age, gender, and smoking
Colak et al., 2011 [9]	European	Cross-sectional	163	NR	66.6	TC, LDL	AREDS	Fundus photography	Total AMD	Low	NR
Ngai et al., 2011 [24]	European	Cohort	949	100.0	71.1	TC, HDL, LDL, TG	WARMGS	Fundus photography	Total AMD	High	Age, systolic BP, CRP, BMI, and blood glucose
Klein et al., 2010 [25]	American	Cross-sectional	2810	45.7	49.0	TC, HDL	WARMGS	Fundus photography	Early AMD	High	Age, gender, and familial correlations
Roh et al., 2008 [26]	Asian	Cross-sectional	9530	56.9	52.9	TC, HDL, LDL, TG	WARMGS	Fundus photography	Total AMD	High	Age and hepatitis B infection
Kawasaki et al., 2008 [27]	Asian	Cross-sectional	1625	44.1	60.4	TC, HDL	WARMGS	Fundus photography	Early and late AMD	High	Age and gender
Cackett et al., 2008 [28]	Asian	Cross-sectional	3280	48.0	58.7	TC, HDL, LDL, TG	WARMGS	Fundus photography	Early and late AMD	High	Age
Tan et al., 2007 [29]	Australian	Cohort	2454	42.4	70.6	TC, HDL, LDL, TG	WARMGS	Fundus photography	Early and late AMD	High	Age, gender, smoking, white cell count, family history of AMD, and very fair skin color
Leeuwen et al., 2004 [30]	European	Cohort	5836	NR	69.6	TC, HDL	WARMGS	Fundus photography	Total AMD	Low	Age, gender, time of follow-up, BMI, smoking, atherosclerosis, alcohol intake, and apolipoprotein E genotype
Klein et al., 2003 [10]	American	Cross-sectional	2361	39.5	78.5	TC, HDL, LDL, TG	WARMGS	Fundus photography	Early AMD	High	Age, gender, and race
Delcourt et al., 2001 [31]	European	Cross-sectional	2584	43.7	70.1	TC, HDL, TG	ICGS	Fundus photography	Early and late AMD	High	Age, gender, smoking, educational level, and plasma α-tocopherol.
Smith et al., 2001 [32]	American/European/Australian	Cross-sectional	14752	42.6	66.0	TC, HDL	WARMGS	Fundus photography	Late AMD	High	Age
Klein et al., 1999 [33]	American	Cross-sectional	8270	48.1	56.3	TC, HDL, TG	WARMGS	Fundus photography	Early AMD	High	Age

AMD, age-related macular degeneration; AREDS, Age-Related Eye Disease Study; BMI, body mass index; CNV, choroidal neovascularization; GA, geographic atrophy; HDL, high-density lipoprotein; ICGS, International Classification and Grading System; LDL, low-density lipoprotein; NR, not reported; TC, total cholesterol; TGs, triglycerides; WARMGS, Wisconsin Age-Related Maculopathy Grading System; * The study quality was judged based on the Newcastle-Ottawa Scale.

**Table 2:** Subgroup analysis of the association between blood lipids and age-related macular degeneration.

Variables	HDL				TC				LDL				TG			
	*n*	RR (95% CI)	*P_H_*	*P_M_*	*n*	RR (95% CI)	*P_H_*	*P_M_*	*n*	RR (95% CI)	*P_H_*	*P_M_*	*n*	RR (95% CI)	*P_H_*	*P_M_*
Any AMD	15	1.18 (1.01, 1.35)	0.007		18	0.96 (0.93, 0.99)	0.001		8	0.93 (0.88, 0.99)	0.83		9	0.91 (0.87, 0.94)	0.42	
Early AMD	11	1.10 (1.01, 1.19)	<0.001		11	0.95 (0.92, 0.99)	0.08		4	0.95 (0.88, 0.99)	0.99		8	0.91 (0.87, 0.95)	0.62	
Late AMD	8	1.14 (0.81, 1.46)	0.25		8	0.97 (0.88, 1.06)	0.56		4	1.00 (0.86, 1.13)	0.30		5	0.96 (0.82, 1.11)	0.34	
GA	3	0.99 (0.23, 1.75)	0.44		3	0.81 (0.62, 1.00)	0.39		-				-			
CNV	3	1.06 (0.51, 1.62)	0.57		3	1.04 (0.86, 1.22)	0.26		-				-			
Design																
Cross-sectional	10	1.23 (0.97, 1.49)	0.01	0.93	14	0.94 (0.87, 1.01)	<0.001	0.86	7	0.93 (0.88, 0.99)	0.73	0.75	8	0.92 (0.89, 0.96)	0.29	0.92
Cohort	5	1.08 (0.93, 1.24)	0.24		5	0.98 (0.93, 1.03)	0.55		1	0.89 (0.17, 1.61)			1	0.68 (0.42, 0.94)		
Ethnicity																
Asian	4	1.17 (0.81, 1.53)	0.49	0.76	6	0.92 (0.81, 1.02)	<0.001	0.78	3	0.95 (0.85, 1.06)	0.91	0.67	3	0.93 (0.83, 1.03)	0.12	0.74
European	6	1.24 (1.10, 1.39)	0.09		7	1.00 (0.92, 1.08)	0.06		4	0.95 (0.87, 1.03)	0.68		4	0.87 (0.77, 0.96)	0.34	
American	4	1.06 (0.55, 1.57)	0.004		4	0.89 (0.76, 1.02)	0.02		1	0.85 (0.73, 0.98)			2	0.90 (0.80, 0.99)	0.12	
Australian	1	0.96 (0.72, 1.20)			1	1.00 (0.91, 1.07)			-				-			
Classification criteria																
WARMGS	11	1.09 (0.91, 1.28)	0.03	0.52	13	0.91 (0.86, 0.97)	0.04	0.54	4	0.95 (0.87, 1.03)	0.91	0.23	5	0.90 (0.85, 0.94)	0.21	0.33
ICGS	4	1.34 (1.13, 1.55)	0.14		4	1.02 (0.97, 1.08)	0.36		3	0.91 (0.82, 0.99)	0.72		4	0.96 (0.90, 1.02)	0.35	
AREDS	-				1	1.89 (1.19, 2.59)			1	1.59 (1.04, 2.14)						
Age (years)																
<65	9	1.06 (0.82, 1.30)	0.03	0.84	10	0.93 (0.87, 1.00)	0.01	0.83	3	0.95 (0.85, 1.06)	0.91	0.55	4	0.94 (0.90, 0.98)	0.23	0.12
≥65	6	1.24 (1.10, 1.39)	0.09		8	0.97 (0.89, 1.06)	<0.001		5	0.92 (0.85, 0.99)	0.54		5	0.86 (0.78, 0.93)	0.47	

AMD, age-related macular degeneration; AREDS, Age-Related Eye Disease Study; BMI, body mass index; CNV, choroidal neovascularization; GA, geographic atrophy; HDL, high-density lipoprotein; ICGS, International Classification and Grading System; LDL, low-density lipoprotein; NR, not reported; *P_H_*: The *p*-value based on Cochran’s Q statistic; *P_M_*: The *p*-value based on meta-regression; RR, relative risk; RS, the Rotterdam Study; TC, total cholesterol; TG, triglycerides; WARMGS, Wisconsin Age-Related Maculopathy Grading System.
